# Minor Hypospadias: The “Tip of the Iceberg” of the Partial Androgen Insensitivity Syndrome

**DOI:** 10.1371/journal.pone.0061824

**Published:** 2013-04-30

**Authors:** Nicolas Kalfa, Pascal Philibert, Ralf Werner, Françoise Audran, Anu Bashamboo, Hélène Lehors, Myriam Haddad, Jean Michel Guys, Rachel Reynaud, Pierre Alessandrini, Kathy Wagner, Jean Yves Kurzenne, Florence Bastiani, Jean Bréaud, Jean Stéphane Valla, Gérard Morisson Lacombe, Mattea Orsini, Jean-Pierre Daures, Olaf Hiort, Françoise Paris, Kenneth McElreavey, Charles Sultan

**Affiliations:** 1 Service de Chirurgie Viscérale et Urologique Pédiatrique, Hôpital Lapeyronie, CHU de Montpellier et Université Montpellier 1, Montpellier, France; 2 Service d'Hormonologie, Hôpital Lapeyronie, CHU de Montpellier et Université Montpellier 1, Montpellier, France; 3 Division of Pediatric Endocrinology and Diabetes, Department of Pediatrics, University of Lübeck, Germany; 4 Human Developmental Genetics Unit, Institut Pasteur, Paris, France; 5 Service de Chirurgie Pédiatrique, Hôpital la Timone, APHM, Marseille, France; 6 Unité d'Endocrinologie et Diabétologie Pédiatriques, Hôpital la Timone, APHM, Marseille, France; 7 Service de Chirurgie Pédiatrique, Hôpital Nord, APHM, Marseille, France; 8 Service de Pédiatrie, Hôpital Lenval, CHU de Nice, France; 9 Service de Chirurgie Pédiatrique, Hôpital Lenval, CHU de Nice, France; 10 Service de Chirurgie Pédiatrique, Hôpital Saint-Jospeh, Marseille, France; 11 Institut Universitaire de Recherche Clinique, Laboratoire de Biostatistiques et d'Epidémiologie, Université Montpellier 1, Montpellier, France; 12 Unité d'Endocrinologie et Gynécologie Pédiatriques, Service de Pédiatrie, Hôpital Arnaud de Villeneuve, CHU de Montpellier et Université Montpellier 1, Montpellier, France; University of North Carolina at Chapel Hill, United States of America

## Abstract

**Background:**

Androgens are critical in male external genital development. Alterations in the androgen sensitivity pathway have been identified in severely undermasculinized boys, and mutations of the androgen receptor gene (*AR*) are usually found in partial or complete androgen insensitivity syndrome (AIS).

**Objective:**

The aim of this study was to determine whether even the most minor forms of isolated hypospadias are associated with *AR* mutations and thus whether all types of hypospadias warrant molecular analysis of the *AR*.

**Materials and Methods:**

Two hundred and ninety-two Caucasian children presenting with isolated hypospadias without micropenis or cryptorchidism and 345 controls were included prospectively. Mutational analysis of the *AR* through direct sequencing (exons 1–8) was performed. In silico and luciferase functional assays were performed for unreported variants.

**Results:**

Five missense mutations of the *AR* were identified in 9 patients with glandular or penile anterior (n = 5), penile midshaft (n = 2) and penile posterior (n = 2) hypospadias, i.e., 3%: p.Q58L (c.173A>T), 4 cases of p.P392S (c.1174C>T), 2 cases of p.A475V (c.1424C>T), p.D551H (c.1651G>C) and p.Q799E (c.2395C>G). None of these mutations was present in the control group. One mutation has never been reported to date (p.D551H). It was predicted to be damaging based on 6 in silico models, and in vitro functional studies confirmed the lowered transactivation function of the mutated protein. Three mutations have never been reported in patients with genital malformation but only in isolated infertility: p.Q58L, p.P392S, and p.A475V. It is notable that micropenis, a cardinal sign of AIS, was not present in any patient.

**Conclusion:**

*AR* mutations may play a role in the cause of isolated hypospadias, even in the most minor forms. Identification of this underlying genetic alteration may be important for proper diagnosis and longer follow-up is necessary to find out if the mutations cause differences in sexual function and fertility later in life.

## Introduction

Hypospadias is defined as a malformation of the penis due to an incomplete development of the ventral part of the penis. This may include (1) a defect in the developing urethra leading to the localization of the urinary meatus on the ventral aspect of the penis in a variable position from the glans to the perineum [1], (2) a defect in the ventral part of the prepuce, and (3) an inconstant ventral penile curvature mainly related to a defect in the ventral skin or, more rarely, the development of the corpus cavernosum. Hypospadias is the second most common congenital malformation in males, occurring in approximately 1 in 125 live male births [2]. In addition to the surgical challenge of correcting this malformation and reducing the non-negligible risk of complications, the clinical challenge today is to elucidate the pathophysiology. A better understanding would optimize childhood management, guide the follow-up of these children to adulthood, and predict those patients at risk of fertility problems in adult life. Unfortunately, the exact etiology remains unknown and is not even sought in most cases, especially anterior isolated hypospadias without any other signs of disorders of sex development such as micropenis or cryptorchidism.

Androgens play a central role in male external genital development. Testosterone and its derivative 5 alpha-dihydrotestosterone are the two major androgens that mediate male sexual differentiation, and an alteration in the androgen sensitivity pathway has been identified in undermasculinized boys [3]. Extensive mutation screening in hypospadiac patients has revealed disease-associated sequence alterations, predominantly in the *AR*. These mutations are usually found in partial or complete androgen insensitivity syndrome [3], [4]. *AR* sequencing is thus generally performed in selected patients with severe 46,XY DSD with normal or elevated plasma level of testosterone [5], but *AR* mutations are usually not considered as a cause of isolated hypospadias [6]–[8], the most minor form of DSD.

It is nevertheless now emerging that many milder variants of the classic disorders exist, especially in partial androgen insensitivity syndrome. Moreover, a systematic approach to genetic analysis is providing rewards in some cases [8], [9]. The aim of this study was to determine whether isolated hypospadias, including the most minor forms, is associated with *AR* mutations and thus whether all types of hypospadias should warrant molecular analysis of the *AR*.

## Materials and Methods

### Patients

In this study, 292 Caucasian boys presenting with isolated hypospadias (no micropenis, no cryptorchidism) were included prospectively (newborn to 12 years). Clinical diagnosis was performed by direct clinical examination by the pediatric urologist or pediatric endocrinologist. The location of the urethral meatus ranged from glandular to perineal hypospadias (glandular and penile anterior n = 190, midshaft n = 61, penile posterior n = 28, penoscrotal and perineal n = 13). The level of division of the corpus spongiosum—which can be assessed during degloving of the penis at the time of surgical correction—was not used as a classification method here since some patients with anterior and glandular hypospadias did not undergo surgery. Three hundred and forty-five controls alleles were sequenced. They included 245 normospermic men [the definition of normospermic is ≥20×10^6^ ml sperm concentration, ≥40×10^6^ total sperm count, ≥2 ml semen volume, ≥50% of a+b or ≥25% motility and a high percentage of normal forms (≥10%) according to the WHO criteria], with normal location of the urethral meatus, noncleaved prepuce and intrascrotal testis, and 50 women of known fertility (more than 2 normal children). This study was approved by the Institutional Review Board of the institution (Centre de Protection des Personnes Sud Méditerannée 4, CPPSMIV, ID RCB n° 2008-A00781-54) and written consent was obtained from all parents.

### DNA extraction

DNA was extracted from either peripheral blood or preputial skin. When blood was used, the DNA was extracted with a QIAamp DNA blood minikit (Qiagen, Courtaboeuf, France). When tissue was used, the excess skin removed at the time of hypospadias surgery and/or circumcision was frozen in liquid nitrogen. DNA was extracted from this tissue using DNAzol (Invitrogen). The manufacturer's protocol for DNA isolation was followed with minor modifications.

### Mutational analysis

After polymerase chain reaction (PCR) amplification of exons 1–8 of the *AR* using the Taq PCR Master Mix kit from Qiagen (Courtaboeuf, France), we performed direct sequencing using the BigDye terminator v1.1 kit (Applied Biosystems, Foster City, CA) and an ABI Prism310 Genetic Analyzer (Applera, Courtaboeuf, France), as reported elsewhere [3]. In cases of mutation, PCR and sequencing of the DNA sample were repeated twice to confirm the finding and rule out any PCR-generated errors. Every PCR product was sequenced with forward and reverse primers. When *AR* mutations were detected, *SRD5A2* and *MAMLD1* genes were also sequenced to rule out another cause of hypospadias [10],[11]. The amino acid numbering for the AR was based on the NCBI reference sequence NM_000044.2 and the AR database [12].

### Homology study and structure prediction

When a mutation that had never been reported was found, the functional consequences of amino acid changes were predicted using in silico models.

Regarding the homology study, ensembl.org detected the putative homologs of the human *AR* gene and alignments were made with the ClustalW software at http://www.ebi.ac.uk/Tools/msa/clustalw2/.

Regarding the structure prediction, the secondary structure for wildtype and variants was predicted using JPred software [13] (http://www.compbio.dundee.ac.uk/www-jpred/). The relative accessibility of amino acids was studied with Netsurf software [14] (http://www.cbs.dtu.dk/services/NetSurfP/). The three-dimensional structure was predicted by the Protein Homology/analogY Recognition Engine (PhyreEngine) from the Structural Bioinformatics Group, Imperial College, London, at http:www.sbg.bio.ic.ac.uk/phyrew/ [15].

The functional consequences of amino acid changes were predicted using three algorithms. Polyphen (Harvard, USA) ([16], [17], Panther {Mi, 2010 #86) and Sift (University of British Columbia) [18] were used, respectively, at http://genetics.bwh.harvard.edu/pph/, http://www.pantherdb.org/tools/csnpScoreForm.jsp., and http://sift.jcvi.org/. These algorithms are based on the alignment of orthologous and/or paralogous protein sequences and/or structural constraints.

### Plasmids

The full-length AR expression construct pSVAR0 was a kind gift of Dr. A. Brinkmann (Rotterdam, NL). The pAR-D551H mutant was constructed by site-directed mutagenesis in a two-step PCR using pSVAR0 as a template. In the first round, the primer pair AR-Kpn_fwd: 5′-CGC ACC TGA TGT GTG GTA CCC T and the mutagenesis primer AR-g1651_as: 5′- GTG GAA AGT AAT AGT GAA TGG GCA AAA CAT GGT CCC T were used in PCR1 and AR-g1651c_s: 5′- AGG GAC CAT GTT TTG CCC ATT CAC TAT TAC TTT CCA C and hARE4a: 5′-ACT ACA CCT GGC TCA ATG GC were used in PCR2. Both amplicons were gel-purified, denatured, annealed and amplified in a second round using primer pair AR-Kpn_fwd and hARE4a. The resulting amplicon was digested with *Kpn*I and *Tth*111I and subcloned into the respective sites of pSVAR0. The construct was verified by sequencing the insert and cloning borders. The PEM-luc firefly luciferase reporter construct containing the proximal promoter of the mouse *Rhox5* gene was a kind gift of Dr. F. Claessens (Leuven, Belgium). The Renilla luciferase construct phRG-TK was obtained from Promega, WI, Madison, USA.

### Cell culture and transfections

Hela cells were maintained in Dulbecco's modified Eagle's medium/Ham's nutrient mixture F-12 (DMEM, Sigma) supplemented with 10% fetal calf serum in 5% CO2 at 37°C.

For transfection, HeLa cells were seeded at 50,000 cells per well in 24-well plates in DMEM medium supplemented with 10% charcoal-stripped fetal calf serum. After 24 hr, cells were transfected with 200 ng of *Rhox5* firefly-luciferase reporter plasmid, 30 ng AR expression plasmid, 10 ng of the constitutive Renilla luciferase expression plasmid phRG-TK, and 0.72 µl Fugene HD (Promega, Madison, WI, USA) per well. Five hours post-transfection, cells were incubated for 18 hr with either vehicle or the indicated concentration DHT, Firefly and Renilla luciferase activities were detected using the Dual-Luciferase reporter assay kit (Promega, Madison, WI, USA) and a LUCY 3 Luminometer (Anthos, Krefeld, Germany). The activity of the Renilla luciferase was used to normalize for transfection efficiency. All transfections were performed in triplicate and in at least 3 independent experiments.

## Results

Five missense mutations of the *AR* were identified in 9 of the 292 patients, i.e., 3%: p.Q58L (c.173A>T), 4 cases of p.P392S (c.1174C>T), 2 cases of p.A475V (c.1424C>T), p.D551H (c.1651 G>C) and p.Q799E (c.2395C>G). None of these mutations was present in the control group. The hypospadias was not severe in 5 cases (glandular and penile anterior n = 5, penile midshaft n = 2) and penile posterior in 2 cases only. Clinical data are summarized in Table 1. Exon 1 was the most frequent mutated exon in this series (n = 7/9). No mutation of *SRD5A2* or *MAMLD1* was found in these patients. The mothers were not available for sequencing.

**Table 1:** Clinical and hormonal data of patients with mutated *AR*.

	p.Q58L (c.1288A>T)	p.P392S (c.2289 C>T)	p.P392S (c.2289 C>T)	p.P392S (c.2289 C>T)	p.P392S (c.2289 C>T)	p.A475V (c.2539 C>T)	p.A475V (c.2539 C>T)	p.D551H (c.1651 G>C)	p.Q799E (c.3510 C>G)
**Medical history**									
Familial history of genital malformation	No	No	Yes, penile hypospadias in brother and cousin on maternal side*	No	No	No	No	No	Yes, penile hypospadias in uncle, maternal side*
Maternal exposure to endocrine disruptors during pregnancy	No	No	No	No	No	No	No	Yes, pesticides	No
Term of birth (weeks of amenorrhea)	41	39	40	39	41	41	40	37	41
Birth weight of (kg)	3.8	4.1	2.7	3.1	3.0	3.7	3.6	3.3	3.2
**Phenotype**									
Age (years, months)	1 y, 7 m	5 y, 6 m	4 y, 4 m	11 y, 9 m	3 y, 7 m	0 y, 7 m	0 y, 7 m	1 week	0 y, 6 m
Weight (kg)	12	24	16	41	16	8.5	8.4	3.3	8
Height (cm)	83	118	NA	152	NA	74	71	48	68
Meatus topography	Glandular	Glandular	Penile midshaft	Penile posterior	Glandular	Penile anterior	Penile midshaft	Penile anterior	Penile posterior
Testis position	Intrascrotal	Intrascrotal	Intrascrotal	Intrascrotal	Intrascrotal	Intrascrotal	Intrascrotal	Intrascrotal	Intrascrotal
Penile length (mm)	33	45	35	33	45	35	50	32	32
Other malformations	No	No	No	High anorectal malformation, right vesicoureteral reflux and mitral insufficiency	No	No	No	No	No
**Hormonal work up**									
Age at hormonal work-up	1 y, 7 m	5 y, 6 m	4 y, 4 m	11 y, 9 m	3 y, 7 m	0 y, 7 m	0 y, 7 m	1 week	0 y, 6 m
FSH (UI/l) (1–10 UI/l)	0.11	0.79	0.11	0.37	0.13	0.69	NA	NA	0.63
LH(UI/l) (1–12 UI/l)	NA	0.1	0.43	1.73	0.467	0.32	NA	NA	2.87
Testosterone (ng/ml) (1–3 ng/ml)	<0.1	<0.1	<0.1	2.26	<0.1	<0.1	0.55	1	1.74

* Family relatives declined genetic examination. NA: not available. Parentheses indicate the normal range for hormone serum levels.

The p.D551H (c.1651 G>C) mutation has never been described and was thus tested in silico.

The secondary structure was predicted to be modified proximal to the mutation with changes in a helical domain of 11 amino acids. The relative and absolute accessibilities of the amino acid were modified from 0.55 to 0.60 and from 77.06 to 110.41, respectively. The structure prediction of the mutated protein was significantly changed (data not shown). All 3 in silico algorithms predicted affected protein function (Polyphen: probably damaging with a 0.99 score; Sift: damaging with a 0.0 score; and Panther: probably a deleterious effect with a 0.79 score) with a conserved amino acid throughout species (table 2).

**Table 2:** Homology study showed that this amino acid was highly conserved through species for the c.1651G>C mutation.

Patient	LETARDHVLPI **H** YYFPPQKTCLI
Human-AR	LETARDHVLPI **D** YYFPPQKTCLI
Pig	LEPTRDHVLPI **D** YYFPPQKTCLI
Chimpanzee	LETARDHVLPI **D** YYFPPQKTCLI
Mouse	LDSTRDHVLPI **D** YYFPPQKTCLI
Rabbit	LETARDHVLPI **D** YYFPPQKTCLI
Dog	LETARDHVLPI **D** YYFPPQKTCLI
Cat	LETSRDHVLPI **D** YYFPPQKTCLI

**Figure 1:**
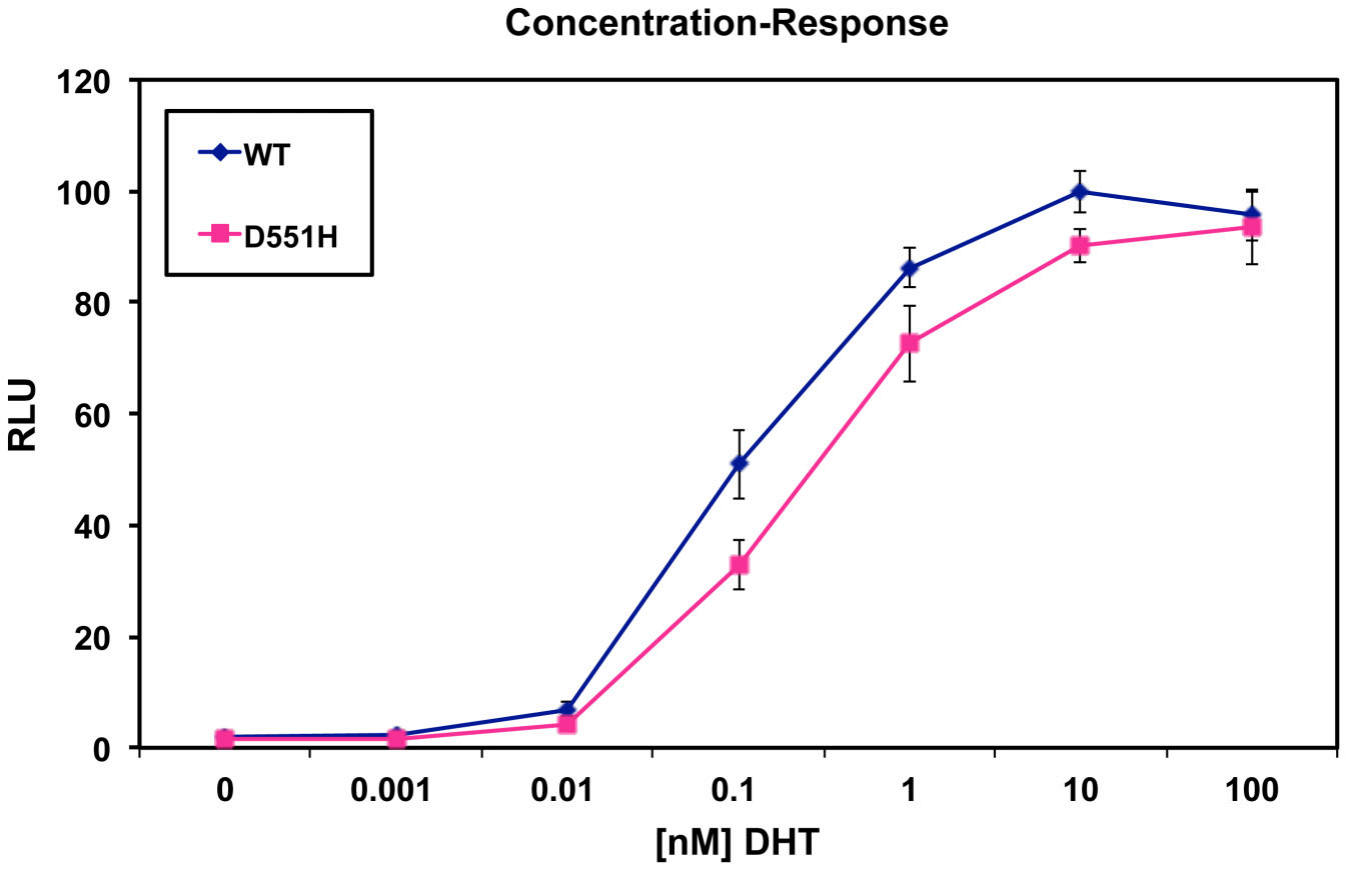
Relative luciferase units (RLU) of the wildtype androgen receptor (WT) and D551H protein and their curves of concentration-response. The D551H mutation significantly reduces the transactivation at DHT concentrations between 0.01 and 10 nM. T-test including equal variance test and was performed using Sigma Stat. *: p = 0.38 ; **: p<0.001.

The in vitro functional studies confirmed that the D551H mutation induced a reduction of the androgen receptor transactivation. The difference between the wild type protein and the mutated one was significant at DHT concentrations between 0.01 and 10 nM (Figure 1).

## Discussion

The recommendations for the appropriate use of genetic testing in male children with genital malformation state that *AR* mutation analysis, along with karyotyping and hormonal work-up, should be performed in children with (1) a phenotype of uncertain sex at birth, (2) severe hypospadias, and (3) hypospadias associated with another sign of DSD that could be a manifestation of partial androgen insensitivity syndrome, such as micropenis or cryptorchidism [19]. However, these recommendations have been based on limited data, and comprehensive studies of a large number of patients with minor hypospadias and complete sequencing of the *AR* including exon 1 remain rare. Vottero et al. [20] recently observed that the *AR* in target tissues from patients with hypospadias is more methylated than in control children, resulting in a decreased expression of the *AR*. However, no mutation was identified. In order to better elucidate the genotype-phenotype relation and identify which patients should be explored, we prospectively screened a large number of unselected hypospadiac subjects.

We found that 3% of 292 boys with isolated hypospadias had *AR* mutations. Two of these mutations have already been associated with severe genital malformation: p.Q799E [21], [22] and p.P392S [23]–[25]. The 3 other mutations may represent novel findings since 1 has never been reported to date (p.D551H, c.1651 G>C) and 2 have not been reported in patients with genital malformation but only isolated infertility: p.Q58L [26], p.A475V [27], [28]. It is notable that micropenis, a cardinal sign of PAIS, was not present in any patient.

The risk of infertility is known to be associated with hypospadias, but there has been no definitive and methodologically adequate study of the fertility of men born with hypospadias. In an evaluation of the social and sexual impact of this malformation, Aho et al. [29] showed that adult men operated on for hypospadias in childhood had fewer children than controls (0.8 vs. 1.1). But multiple factors such as surgical results, psychological aspects and quality of ejaculation may have contributed to this observation. The semen of corrected hypospadiac patients (isolated or not) was tested by Bracka [30] and 30% of these patients had sperm counts below 20 million/ml. Unfortunately, the development of spermatogenesis in children born with hypospadias remains little documented due to the difficulty of long term follow-up, the absence of a univocal etiology of this malformation, and the multiplicity of factors that may influence male fertility. The association of hypospadias with other testicular developmental abnormalities, especially cryptorchidism, raises further questions about the future fertility of these patients. Cryptorchidism is more frequent in these patients than in the general population [31], but the rate is similar to the 5% to 10% found in infertile men [32].

Thus, the patients with the highest risk are mainly those with cryptorchidism and those with a severe meatus displacement [33]. In contrast, children with anterior and isolated hypospadias are thought to be as fertile as the general population. Our findings, along with the possibly altered hormonal work-up in these patients [34], raise questions about this unproven dogma. Approximately 40% of the men with isolated hypospadias have a sperm concentration below 40 million/ml, which may indicate subfecundity [35], [36]. A study reporting the histological aspect of testicular biopsies from 33 patients who had been operated on for hypospadias in childhood also point toward the risk of infertility in patients with isolated hypospadias [37]. Twenty-seven percent of them had an abnormal testicular histology suggesting low spermatogenesis, compared with 75% of patients with hypospadias and cryptorchidism. The finding of *AR* mutations even in the most minor forms of hypospadias may indicate that these mutations make up part of the common background of these two diseases. Early detection of the *AR* mutations known to be usually present in patients with PAIS syndromes or hypofertile men might also significantly improve (1) the hormonal and clinical follow-up of these patients during puberty, especially regarding the size of the penis. Indeed, the activities and effects of androgens during fetal life do not always predict their impact on penile growth during puberty [38]; and (2) the information given to parents and patients: identifying the genetic origin of this malformation might help to provide adequate fertility information and genetic counseling for the daughters.

It is notable that 7 out of 9 patients exhibited a mutation in exon 1 of the *AR*, whereas about 85% of the mutations reported in the *AR* mutations database [39] (http://www.mcgill.ca/androgendb/) are localized in exons 2 to 8. In contrast, the mutations responsible for minor phenotypes, as seen in our series, are mainly present in exon 1. Of the exon 1 mutations described in the *AR* database, 70% induced complete androgen insensitivity syndrome (CAIS), 14% induced partial androgen insensitivity syndrome (PAIS), and 16% were implicated in infertility. Exon 1 is an important regulatory region for AR activity. It encodes the N-terminal domain of the AR protein, which contains transactivation elements, dimerization zones, several cofactor binding sites, and two regions (^25^FXXLF^27^ and ^433^WXXLF^437^) involved in N-terminal and C-terminal domain interactions. Loss of this domain results in inactivation of the AR since testosterone binding no longer leads to its conformational change and the subsequent interactions between helix H12 and helix H3 [40]. Exon 1 mutations are thus mainly associated with CAIS. In a recent report by Philibert et al. [3], all these mutations led to a premature stop codon and totally disrupted AR transductional activity because the protein was truncated, explaining the severe phenotype. In our study, the phenotype was minor with isolated hypospadias and no nonsense mutation was identified. The functional studies of the D551H mutation demonstrating a slightly lowered transactivation of the receptor are in agreement with some level of genotype-phenotype correlation. Such a correlation has not been demonstrated for the AR and there are important variations in phenotypes for a single *AR* mutation in the database (ranging from PAIS to infertility). These results nevertheless show that missense mutations of exon 1 may slightly alter AR function, inducing minor phenotypes.

Isolated and minor hypospadias is the most frequent form of 46,XY DSD, and screening all patients with this phenotype would be very expensive and time-consuming. It would thus be helpful to identify the subgroup of these children who need to be screened for an *AR* mutation. In our series, no clinical data from the medical history or physical examination were able to identify patients at risk for these mutations. Mutated subjects had no other signs of androgen insensitivity than isolated hypospadias. Size of the penis, location of testes and location of the urethral meatus were unable to identify *a priori* patients with a higher risk of AR abnormalities. Familial history was more frequent in patients with an *AR* mutation (2/7) than in the others (28.5% vs 13%, p>0.05), but this criterion is not sufficient and it misses most of the patients with a genetic alteration. The clinical data alone cannot be used as indicators of *AR* mutation. The hormonal work-up was of no help either. Although higher LH and testosterone concentrations are observed in patients with more severe forms of androgen insensitivity such as CAIS, measurements of LH, FSH and plasma testosterone were normal or inconclusive in our series since most patients with a minor phenotype are referred to the surgeon during infancy, far after the neonatal period of pituitary-testicular activity (mini-puberty). Conducting systematic HCG tests in these patients would be abusive.

## Conclusion

*AR* mutations may play a role in the cause of isolated hypospadias, even in the most minor forms of this malformation. We found a prevalence of mutations of about 3%. These patients with AR abnormalities did not differ from the vast majority of hypospadiac boys. Identification of the underlying alteration in the *AR* may be important for a proper diagnosis of this frequent genital abnormality. Longer follow-up of these patients is necessary to determine whether these mutations cause differences in sexual function and fertility later in life.

## References

[1] Leung AK, Robson WL (2007) Hypospadias: an update. Asian J Androl 9: 16–22. 17187155 10.1111/j.1745-7262.2007.00243.x

[2] Manson JM, Carr MC (2003) Molecular epidemiology of hypospadias: review of genetic and environmental risk factors. Birth Defects Res A Clin Mol Teratol 67: 825–836. 14745936 10.1002/bdra.10084

[3] Philibert P, Audran F, Pienkowski C, Morange I, Kohler B, et al (2010) Complete androgen insensitivity syndrome is frequently due to premature stop codons in exon 1 of the androgen receptor gene: an international collaborative report of 13 new mutations. Fertil Steril 94: 472–476. 19463997 10.1016/j.fertnstert.2009.03.057

[4] Sultan C, Paris F, Terouanne B, Balaguer P, Georget V, et al (2001) Disorders linked to insufficient androgen action in male children. Hum Reprod Update 7: 314–322. 11392378 10.1093/humupd/7.3.314

[5] Deeb A, Mason C, Lee YS, Hughes IA (2005) Correlation between genotype, phenotype and sex of rearing in 111 patients with partial androgen insensitivity syndrome. Clin Endocrinol (Oxf) 63: 56–62. 15963062 10.1111/j.1365-2265.2005.02298.x

[6] Nordenskjold A, Friedman E, Tapper-Persson M, Soderhall C, Leviav A, et al (1999) Screening for mutations in candidate genes for hypospadias. Urol Res 27: 49–55. 10092153 10.1007/s002400050088

[7] Muroya K, Sasagawa I, Suzuki Y, Nakada T, Ishii T, et al (2001) Hypospadias and the androgen receptor gene: mutation screening and CAG repeat length analysis. Mol Hum Reprod 7: 409–413. 11331662 10.1093/molehr/7.5.409

[8] Wang Y, Li Q, Xu J, Liu Q, Wang W, et al (2004) Mutation analysis of five candidate genes in Chinese patients with hypospadias. Eur J Hum Genet 12: 706–712. 15266301 10.1038/sj.ejhg.5201232

[9] Silver RI, Russell DW (1999) 5alpha-reductase type 2 mutations are present in some boys with isolated hypospadias. J Urol 162: 1142–1145. 10458450 10.1016/S0022-5347(01)68102-3

[10] Maimoun L, Philibert P, Cammas B, Audran F, Bouchard P, et al (2011) Phenotypical, biological, and molecular heterogeneity of 5alpha-reductase deficiency: an extensive international experience of 55 patients. J Clin Endocrinol Metab 96: 296–307. 21147889 10.1210/jc.2010-1024

[11] Kalfa N, Cassorla F, Audran F, Oulad Abdennabi I, Philibert P, et al (2011) Polymorphisms of MAMLD1 gene in hypospadias. J Pediatr Urol 7: 585–591. 22030455 10.1016/j.jpurol.2011.09.005

[12] Gottlieb B, Beitel LK, Nadarajah A, Paliouras M, Trifiro M (2012) The androgen receptor gene mutations database: 2012 update. Hum Mutat 33: 887–894. 22334387 10.1002/humu.22046

[13] Cole C, Barber JD, Barton GJ (2008) The Jpred 3 secondary structure prediction server. Nucleic Acids Res 36: W197–201. 18463136 10.1093/nar/gkn238 PMC2447793

[14] Petersen B, Petersen TN, Andersen P, Nielsen M, Lundegaard C (2009) A generic method for assignment of reliability scores applied to solvent accessibility predictions. BMC Struct Biol 9: 51. 19646261 10.1186/1472-6807-9-51 PMC2725087

[15] Kelley LA, Sternberg MJ (2009) Protein structure prediction on the Web: a case study using the Phyre server. Nat Protoc 4: 363–371. 19247286 10.1038/nprot.2009.2

[16] Ramensky V, Bork P, Sunyaev S (2002) Human non-synonymous SNPs: server and survey. Nucleic Acids Res 30: 3894–3900. 12202775 10.1093/nar/gkf493 PMC137415

[17] Thomas PD, Kejariwal A (2004) Coding single-nucleotide polymorphisms associated with complex vs. Mendelian disease: evolutionary evidence for differences in molecular effects. Proc Natl Acad Sci U S A 101: 15398–15403. 15492219 10.1073/pnas.0404380101 PMC523449

[18] Kumar P, Henikoff S, Ng PC (2009) Predicting the effects of coding non-synonymous variants on protein function using the SIFT algorithm. Nat Protoc 4: 1073–1081. 19561590 10.1038/nprot.2009.86

[19] Edery P (2007) Etiological Aspects of Hypospadias. Dialogues in Pediatric Urology 28: 1–15.

[20] Vottero A, Minari R, Viani I, Tassi F, Bonatti F, et al (2012) Evidence for epigenetic abnormalities of the androgen receptor gene in foreskin from children with hypospadias. J Clin Endocrinol Metab 96: E1953–1962. 10.1210/jc.2011-0511 21937623

[21] Bevan CL, Brown BB, Davies HR, Evans BA, Hughes IA, et al (1996) Functional analysis of six androgen receptor mutations identified in patients with partial androgen insensitivity syndrome. Hum Mol Genet 5: 265–273. 8824883 10.1093/hmg/5.2.265

[22] Quigley CA, De Bellis A, Marschke KB, el-Awady MK, Wilson EM, et al (1995) Androgen receptor defects: historical, clinical, and molecular perspectives. Endocr Rev 16: 271–321. 7671849 10.1210/edrv-16-3-271

[23] Hiort O, Holterhus PM, Horter T, Schulze W, Kremke B, et al (2000) Significance of mutations in the androgen receptor gene in males with idiopathic infertility. J Clin Endocrinol Metab 85: 2810–2815. 10946887 10.1210/jcem.85.8.6713

[24] Bhangoo A, Paris F, Philibert P, Audran F, Ten S, et al (2010) Isolated micropenis reveals partial androgen insensitivity syndrome confirmed by molecular analysis. Asian J Androl 12: 561–566. 20305676 10.1038/aja.2010.6 PMC3739378

[25] Audi L, Fernández-Cancio M, Carrascosa A, Andaluz P, Torán N, et al (2010) Novel (60%) and recurrent (40%) androgen receptor gene mutations in a series of 59 patients with a 46,XY disorder of sex development. J Clin Endocrinol Metab 95: 1876–1888. 20150575 10.1210/jc.2009-2146

[26] Lund A, Juvonen V, Lahdetie J, Aittomaki K, Tapanainen JS, et al (2003) A novel sequence variation in the transactivation regulating domain of the androgen receptor in two infertile Finnish men. Fertil Steril 79 Suppl 3: 1647–1648. 12801573 10.1016/s0015-0282(03)00256-5

[27] Zuccarello D, Ferlin A, Vinanzi C, Prana E, Garolla A, et al (2008) Detailed functional studies on androgen receptor mild mutations demonstrate their association with male infertility. Clin Endocrinol (Oxf) 68: 580–588. 17970778 10.1111/j.1365-2265.2007.03069.x

[28] Ferlin A, Vinanzi C, Garolla A, Selice R, Zuccarello D, et al (2006) Male infertility and androgen receptor gene mutations: clinical features and identification of seven novel mutations. Clin Endocrinol (Oxf) 65: 606–610. 17054461 10.1111/j.1365-2265.2006.02635.x

[29] Aho MO, Tammela OK, Somppi EM, Tammela TL (2000) Sexual and social life of men operated in childhood for hypospadias and phimosis. A comparative study. Eur Urol 37: 95–100. 10671793 10.1159/000020107

[30] Bracka A (1989) A long-term view of hypospadias. Br J Plast Surg 42: 251–255. 2758195 10.1016/0007-1226(89)90140-9

[31] Thonneau PF, Gandia P, Mieusset R (2003) Cryptorchidism: incidence, risk factors, and potential role of environment; an update. J Androl 24: 155–162. 12634298 10.1002/j.1939-4640.2003.tb02654.x

[32] Mieusset R, Bujan L, Massat G, Mansat A, Pontonnier F (1995) Clinical and biological characteristics of infertile men with a history of cryptorchidism. Hum Reprod 10: 613–619. 7782441 10.1093/oxfordjournals.humrep.a135998

[33] Asklund C, Jensen TK, Main KM, Sobotka T, Skakkebaek NE, et al (2009) Semen quality, reproductive hormones and fertility of men operated for hypospadias. Int J Androl 33: 80–87. 19281491 10.1111/j.1365-2605.2009.00957.x

[34] Rey RA, Codner E, Iniguez G, Bedecarras P, Trigo R, et al (2005) Low risk of impaired testicular Sertoli and Leydig cell functions in boys with isolated hypospadias. J Clin Endocrinol Metab 90: 6035–6040. 16131574 10.1210/jc.2005-1306

[35] Slama R, Eustache F, Ducot B, Jensen TK, Jorgensen N, et al (2002) Time to pregnancy and semen parameters: a cross-sectional study among fertile couples from four European cities. Hum Reprod 17: 503–515. 11821304 10.1093/humrep/17.2.503

[36] Guzick DS, Overstreet JW, Factor-Litvak P, Brazil CK, Nakajima ST, et al (2001) Sperm morphology, motility, and concentration in fertile and infertile men. N Engl J Med 345: 1388–1393. 11794171 10.1056/NEJMoa003005

[37] Jugenburg I, Kipikasa A (1988) Fertility in patients with hypospadias. Acta Chir Plast 30: 86–93. 2470226

[38] Dean A, Smith LB, Macpherson S, Sharpe RM (2012) The effect of dihydrotestosterone exposure during or prior to the masculinization programming window on reproductive development in male and female rats. Int J Androl 35: 330–339. 22248293 10.1111/j.1365-2605.2011.01236.x

[39] Gottlieb B, Beitel LK, Wu JH, Trifiro M (2004) The androgen receptor gene mutations database (ARDB): 2004 update. Hum Mutat 23: 527–533. 15146455 10.1002/humu.20044

[40] He B, Kemppainen JA, Wilson EM (2000) FXXLF and WXXLF sequences mediate the NH2-terminal interaction with the ligand binding domain of the androgen receptor. J Biol Chem 275: 22986–22994. 10816582 10.1074/jbc.M002807200

